# *Leea indica* Ethyl Acetate Fraction Induces Growth-Inhibitory Effect in Various Cancer Cell Lines and Apoptosis in Ca Ski Human Cervical Epidermoid Carcinoma Cells

**DOI:** 10.1155/2011/293060

**Published:** 2011-03-01

**Authors:** Wong Yau Hsiung, Habsah Abdul Kadir

**Affiliations:** Biomolecular Research Group, Biochemistry Division, Institute of Biological Sciences, Faculty of Science, University of Malaya, 50603 Kuala Lumpur, Malaysia

## Abstract

The anticancer potential of *Leea indica*, a Chinese medicinal plant was investigated for the first time. The crude ethanol extract and fractions (ethyl acetate, hexane, and water) of *Leea indica* were evaluated their cytotoxicity on various cell lines (Ca Ski, MCF 7, MDA-MB-435, KB, HEP G2, WRL 68, and Vero) by MTT assay. *Leea indica* ethyl acetate fraction (LIEAF) was found showing the greatest cytotoxic effect against Ca Ski cervical cancer cells. Typical apoptotic morphological changes such as DNA fragmentation and chromatin condensation were observed in LIEAF-treated cells. Early signs of apoptosis such as externalization of phosphatidylserine and disruption of mitochondrial membrane potential indicated apoptosis induction. This was further substantiated by dose- and time-dependent accumulation of sub-G_1_ cells, depletion of intracellular glutathione, and activation of caspase-3. In conclusion, these results suggested that LIEAF inhibited cervical cancer cells growth by inducing apoptosis and could be developed as potential anticancer drugs.

## 1. Introduction

Apoptosis is a physiological cell suicide program which can be evoked in response to stimuli such as ionizing radiation, toxins, and anticancer drugs. The induction of apoptosis is known to be an efficient and promising strategy to kill cancer cells [1]. Many natural plants extracts and phytochemicals have been reported to induce apoptosis in cancer cell lines [2–4]. In the present study, *Leea indica*, a traditional Chinese medicinal plant was selected for the evaluation of its anticancer potential.

*Leea indica* (Burm. f.) Merr. (Leeaceae) is a shrub which can be found in tropical and subtropical countries including Malaysia, Thailand, and China. It is commonly known as memali (in Malay) and yantuo (in Chinese) and claimed to have some medicinal values such as anticancer, antidiabetic, antidiarrhoeal, antidysenteric, and antispasmodic based on local uses [5–8]. Previous biological studies have shown that it possessed strong antioxidant, phosphodiesterase and nitric oxide synthase inhibitory activities [9, 10]. Apart from the initial screening against breast cancer cell line [11], there is no report showing the cytotoxicity of *Leea indica* on other cancer cell lines. Moreover, no detailed mechanism of action underlying the cytotoxicity of *Leea indica* had been delineated. In view of that, it was thus necessary to expand the present study to include the cytotoxicity on different cancer cell lines and the possible mechanisms underlying the cytotoxicity action.

## 2. Methods

### 2.1. Preparation of Crude Ethanol Extract and Fractions from *Leea indica*

The fresh leaves of *Leea indica* were collected from Ipoh, Perak, and Malaysia and were authenticated by Professor Dr. Ong Hean Chooi, a botanist from the Institute of Biological Sciences, Faculty of Science, University of Malaya, Malaysia. The leaves of *Leea indica* (10 kg) were washed, oven-dried for one week at a constant temperature of 50°C, and ground to a fine powder using a dry grinder. The dried, ground leaf powder (3.86 kg) was then soaked in ethanol for 3 days at room temperature. The extract was then separated from the residue by filtration through Whatman No. 1 filter paper. The remaining residue was re-extracted twice with ethanol. The filtrate from each extraction was combined and the solvent was removed under reduced pressure at 40°C using a rotary evaporator (Buchi) to give a dark green crude ethanol extract (168.53 g). A portion of the ethanol extract (150 g) was further extracted with hexane to give a hexane soluble fraction (10.95 g) and the hexane-insoluble residue was further partitioned between ethyl acetate and water to yield an ethyl acetate-soluble fraction (96.03 g). The water layer was freeze-dried to give a water fraction (20.53 g). The *Leea indica* ethanol extract (LIEE),* Leea indica* ethyl acetate fraction (LIEAF),* Leea indica* hexane fraction (LIHF) and* Leea indica* water fraction (LIWF) were dissolved in dimethyl sulfoxide (DMSO) prior to each assay. The final concentration of DMSO in all the experiments did not exceed 0.5% v/v. All samples were filter sterilized with 0.22 *μ*m filters before use.

### 2.2. Cell Culture

Ca Ski (cervical epidermoid carcinoma cells), MCF 7 (breast carcinoma cells), MDA-MB-435 (melanoma cells), KB (nasopharyngeal epidermoid carcinoma cells, HeLa derivative) HEP G_2_ (hepatocellular carcinoma cells), WRL 68 (liver embryonic cells, HeLa derivative) and Vero (kidney epithelial cells) were obtained from the American Type Culture Collection (ATCC, USA). The MDA-MB-435 cell line was originally thought to be breast cancer cell but recent reports showed that it was melanoma cell line [12, 13]. The KB and WRL 68 cell lines which previously thought to be nasopharyngeal epidermoid carcinoma cell and embryonic liver cell, respectively, were actually derivative of Hela cervical carcinoma cells [14–17]. KB cells were maintained in Medium 199 (Sigma), WRL 68 cells in DMEM (Dulbecco's Modified Eagle's Medium) (Sigma), HEP G2 and Vero in EMEM (Eagle's Minimum Essential Medium) (Sigma), and Ca Ski, MCF 7, and MDA-MB-435 cells in RPMI 1640 Medium (Sigma). All the media were supplemented with 10% (v/v) heat-inactivated fetal bovine serum (PAA Lab, Austria), 100 *μ*g/mL streptomycin and 100 unit/mL penicillin (PAA Lab, Austria), and 50 *μ*g/mL amphotericin B (PAA Lab, Austria). The media were filter sterilized using a 0.22 *μ*m filter membrane (Minisart, Sartorius stedim). The cells were cultured in 5% CO_2_ incubator at 37°C in a humidified atmosphere. The culture was subcultured every 2 or 3 days and routinely checked under an inverted microscope (Motic) for any contamination. Cells in the exponential growth phase were used for all experiments. The cells were harvested from culture flasks by Accutase (Innovative Cell Technologies) and the viable cell count was determined using trypan blue exclusion assay with a hemocytometer.

### 2.3. MTT Cytotoxicity Assay

The cell viability was investigated using MTT [3-(4,5-dimethylthiazol-2-yl)-2,5-diphenyltetrazolium bromide] assay [18]. Viable cells were seeded into 96-well flat-bottomed culture plates and allowed to adhere overnight and then treated with various concentrations (10–200 *μ*g/mL) of *Leea indica* extract and fractions. For the untreated cells (control), vehicle dimethyl sulfoxide (DMSO) was added instead of the sample. After 72-hour incubation, 20 *μ*L MTT (5 mg/mL) (Sigma) was added to each well, and the plates were incubated for another 4 hours at 37°C. Following incubation, the culture medium was removed by gentle aspiration and replaced with 150 *μ*L DMSO, to dissolve the formazan crystals. The amount of formazan product was measured at 570 nm and 650 nm as a background using a microplate reader (Oasys UVM340). The percentage of cell viability equals (absorbance of treated cells/absorbance of untreated cells) × 100%.

### 2.4. Morphological Detection of Apoptosis Using DAPI Nuclear Stain

The occurrence of apoptosis in Ca Ski cells was evaluated using 4′, 6-diamidino-2-phenylindole (DAPI, Sigma) staining. In brief, Ca Ski cells were grown on cover slips and then treated with LIEAF. After the indicated period, the cells were washed with PBS and fixed with acetone at −20°C for 30 minutes. The cells were then stained with DAPI solution (1 *μ*g/mL) at 4°C for 30 minutes. The cover slips were then mounted onto glass microscope slides and observed under fluorescence microscopy (Nikon) using a 350 nm excitation and a 460 nm emission fluorescent filter.

### 2.5. Cell Cycle Analysis

The DNA content and cell cycle distribution were assessed using propidium iodide (PI) staining. LIEAF-treated Ca Ski cells were collected, washed with PBS, and fixed with ice-cold absolute ethanol at −20°C overnight. Fixed cells were then washed and resuspended in staining buffer containing 50 *μ*g/mL PI, 0.1% sodium citrate, 0.1% Triton-X-100, and 100 *μ*g/mL RNase A. The cell suspensions were incubated at room temperature in darkness for 1 hour and analyzed by flow cytometry.

### 2.6. Assessment of Early and Late Apoptosis Using Annexin V/PI Staining

After treatment with LIEAF, both floating and adherent cells were harvested, washed twice with PBS, resuspended in annexin V binding buffer (BD), and stained with annexin V-FITC and PI (BD) at room temperature in the dark for 30 minutes. The cells were then analyzed by flow cytometry. Annexin V was used to detect both the early and late stages of apoptosis while PI was used to detect late apoptosis and necrosis. The discrimination between viable (both annexin V and PI negative), early apoptotic (annexin V positive and PI negative), late apoptotic (both annexin V and PI positive), and necrotic (annexin V negative and PI positive) cells was achieved by quantitatively estimating the relative amounts of the annexin V/PI-stained cells in the population.

### 2.7. Flow Cytometric Measurement of Mitochondrial Membrane Potential (MMP)

The change in MMP was assessed using the cell-permeable, mitochondrial-specific fluorescent probe JC-1 [19]. After treatment with LIEAF, the cells were harvested, washed, and resuspended in medium containing JC-1 (BD). Then the cells were incubated at 37°C in the CO_2_ incubator for 15 minutes. Next, the cells were washed again and resuspended in the medium. Finally, the cells were subjected to flow cytometry analysis by detecting the green and red fluorescence signals. Healthy cells which contained the JC-1 aggregates (red fluorescence) within the mitochondria were detected in the FL-2 channel. Meanwhile, apoptotic cells which contained the JC-1 monomers (green fluorescence) in the cytoplasm were detected in the FL-1 channel. For fluorescence microscopy analysis, the cells were grown on the cover slips and treated with LIEAF. At the end of treatment period, the medium was removed and replaced with JC-1 medium. Then the cells were incubated for 15 minutes. The cover slips were then mounted on the microscope slides and observed under fluorescence microscope.

### 2.8. Terminal Deoxynucleotidyl Transferase dUTP Nick End Labeling (TUNEL) Assay

A TUNEL assay kit (Sigma) was selected to detect DNA fragmentation by labeling the terminal end of nucleic acids. The procedure was based on the manufacturer protocol. In brief, LIEAF-treated Ca Ski cells were harvested, washed with PBS, and fixed with 1% (w/v) paraformaldehyde in ice-cold PBS for 15 minutes. After fixation, the cells were washed and incubated in DNA labeling solution for 60 minutes at 37°C. The cells were then rinsed and incubated with buffer containing FITC-labeled anti-BrdU antibody for 30 minutes at room temperature. Then PI/RNase A solution was added and the cells were further incubated at room temperature in darkness for 30 minutes and analyzed by flow cytometry.

### 2.9. Determination of Intracellular Total Glutathione (GSH) Content

After treatment, the cells were harvested and collected by centrifugation, washed with ice-cold PBS, resuspended in 500 *μ*L of 5% 5-sulfosalicylic acid, and incubated on ice for 15 minutes with intermittent vortexing. The suspension was then centrifuged at 10000 rpm for 15 minutes to collect the supernatant. The supernatant was then subjected to glutathione assay in 96-well plate format whereby to each well were added GSH standards, 5,5-dithio-bis(2-nitrobenzoic acid) (DTNB), and NADPH in phosphate buffer. The reaction was started by quickly adding glutathione reductase. The final concentrations of these components in the reaction mixture were 95 mM potassium phosphate buffer (pH 7.0), 0.95 mM EDTA, 0.038 mg/mL (48 *μ*M) NADPH, 0.031 mg/mL DTNB, 0.115 units/mL glutathione reductase, and 0.24% 5-sulfosalicylic acid. Absorbance was read at 30-second intervals for 10 minutes at 405 nm with a Oasys UVM340 microplate reader and compared with a glutathione standard curve.

### 2.10. Caspase-3 Activity Assay

Caspase-3 activity was measured using the Caspase-3 colorimetric detection kit (Sigma) according to the recommended protocol based on the cleavage of a specific colorigenic substrate, Ac-DEVD-pNA. Cells were seeded in sterile 60mm dishes, and at end of LIEAF treatment, the cells were washed with PBS and lysed in lysis buffer provided by the kit. After freezing and thawing three times, the cell lysate was centrifuged at 20,000x g at 4°C for 15 minutes. The supernatants were collected and Ac-DEVD-pNA was then added and incubated for overnight at 37°C. The concentration of the pNA released was measured at 405 nm, and the quantity of pNA was calculated from a calibration curve of pNA standard. Caspase-3 activity was expressed as fold increase compared to the control untreated cells. For the study of the effect of caspase-3 inhibitor on LIEAF-induced apoptosis, Ca Ski cells were pretreated with 50 *μ*M of Ac-DEVD-CHO, a caspase-3 specific inhibitor for 1 hour before being treated with LIEAF. Inhibition of apoptosis by Ac-DEVD-CHO was further evaluated by TUNEL assay, PI, and annexin V-PI staining as described in method.

### 2.11. Statistical Analysis

In all the experiments, data were expressed as means ± standard error. A significant difference from the respective control for each experiment was assessed using Student's *t*-test, with *P* values <  .05 being regarded as statistically significant.

## 3. Results

### 3.1. Dose-Dependent Reduction of Ca Ski Cells Viability by LIEAF

**Figure 1:**
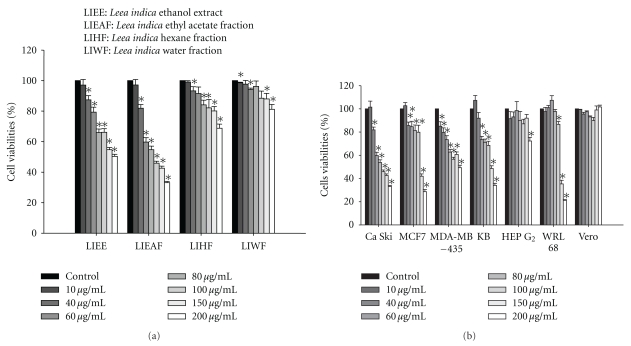
(a) Effect of *Leea indica* extract and fractions on the viability of Ca Ski cells. Ca Ski cells were treated with vehicle DMSO (untreated control) or with increasing concentrations (10–200 *μ*g/mL) of extract and fractions of* Leea indica *(LIEE, LIEAF, LIHF and LIWF) for 72 hours and the cell viability was assessed by MTT assay. (b) Effect of LIEAF on the viability of different cell lines. Each cell line was treated with vehicle DMSO (untreated control) or increasing concentrations (10–200 *μ*g/mL) of LIEAF for 72 hours and the cells viability was determined by MTT assay. Results were expressed as mean percentages of cell viability (ratio of absorbance in treated cells to untreated control cells) ± S.E. of three individual experiments. The asterisks represented significantly different value from control (*P < .05).

The *in vitro* cytotoxic effect of *Leea indica* was evaluated by MTT assay. The results showed that the crude ethanol and fractions of *Leea indica* significantly reduced the viability of Ca Ski cells in a dose-dependent manner (Figure 1(a)). Among the fractions, LIEAF appeared to demonstrate the greatest growth inhibitory effect against Ca Ski cells, followed by LIEE, LIHF, and LIWF. The IC_50_ values (concentration that reduces cell viability to 50%), in ascending order, were 85.83 ± 6.01 *μ*g/mL, 188.03 ± 2.875 *μ*g/mL, >200 *μ*g/mL and >200 *μ*g/mL for LIEAF, LIEE, LIHF, and LIWF, respectively. Furthermore, LIEAF also showed significant dose-dependent reduction of cell viability on MCF 7 (IC_50_ = 138.05 ± 19.16 *μ*g/mL), KB (IC_50_ = 146.9 ± 10.41 *μ*g/mL), and MDA-MB-435 (IC_50_ > 200 *μ*g/mL) while for HEP G2 and WRL 68 cells, significant decrease of cell viability was only observed at 100 *μ*g/mL and above. However, LIEAF showed no effect on the viability of Vero cells (Figure 1(b)). 

### 3.2. Elicitation of Apoptotic Nuclear Morphological Changes by LIEAF

**Figure 2:**
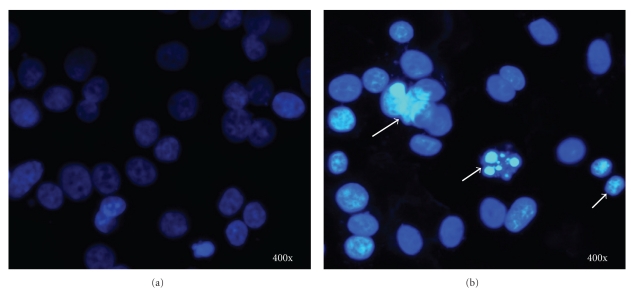
Effect of LIEAF on the nuclear morphology of Ca Ski cells. Ca Ski cells were incubated in the absence (a) or presence of 500 *μ*g/mL LIEAF for 24 hours (b). The cells were stained with DAPI and examined by fluorescent microscopy. Arrows showed signs of nuclear shrinkage and chromatin condensation. Magnification is 400x.

DAPI staining demonstrated that LIEAF elicited nuclear morphological changes characteristic of apoptosis in Ca Ski cells. In the control-untreated group (Figure 2(a)), the cells were in rounded shape and the large nuclei were homogenously stained with a less bright blue color; however, after treatment with 500 *μ*g/mL of LIEAF for 24 hours, the blue emission light was much brighter and condensed than the control cells. Furthermore, signs of nuclear shrinkage and chromatin condensation (Figure 2(b)) were also observed. 

### 3.3. Dose- and Time-Dependent Induction of Sub-G_1_ Cells by LIEAF

**Figure 3:**
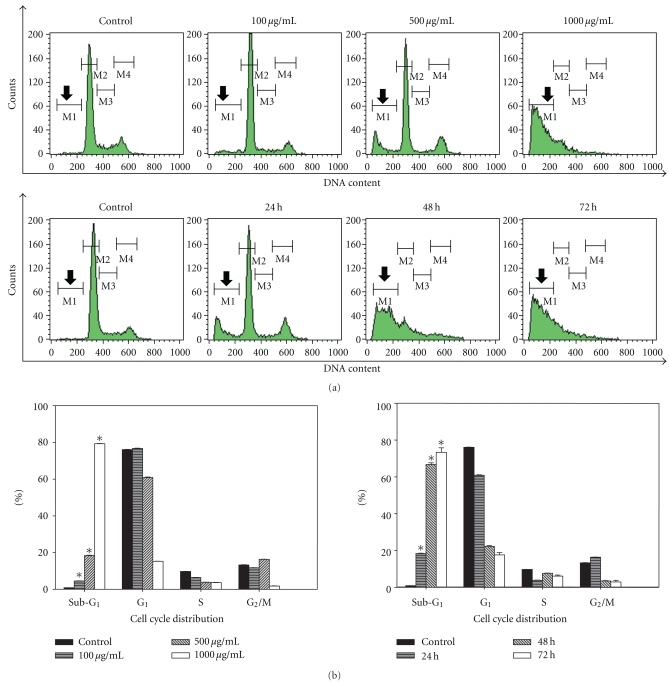
Effect of LIEAF on the cell cycle distribution of Ca Ski cells. Ca Ski cells were incubated in the absence (control) or presence of 100, 500, and 1000 *μ*g/mL of LIEAF for 24 hours. In another experiment, Ca Ski cells were incubated in the absence (control) or presence of 500 *μ*g/mL of LIEAF for 24, 48, and 72 hours. The cells were then stained with PI solution and analyzed by flow cytometer. (a) Histograms obtained from PI staining. The region M1 shown by the arrows is corresponding to cells with sub-G_1_ DNA content. (b) Bar charts showed the cell cycle distribution upon LIEAF treatment. The asterisk represented significantly different value from control (*P < .05). The data represent mean ± S.E. of three different experiments.

To further demonstrate that the inhibition of cell growth could be accompanied by alterations in cell cycle distribution, the cells were treated with LIEAF and stained with PI. The percentage of cells in different stages of the cell cycle was analyzed using flow cytometer. The results showed a significant increase of sub-G_1_ cells, as revealed by the sub-G_1_  peak (Figure 3(a)), in a dose-dependent manner, accounting for 4.54 ± 0.07%, 18.40 ± 0.19%, and 79.24 ± 0.16% of the population after treatment with 100, 500, and 1000 *μ*g/mL of LIEAF, respectively, for 24 hours. This was accompanied by a decrease of G_1_ and S phase cells at increasing doses (Figure 3(b)). Similarly, treatment with 500 *μ*g/mL of LIEAF for 24, 48, and 48 hours also led to a signifcant time-dependent increase of sub-G_1_ cells (18.40 ± 0.19%, 66.75 ± 0.93%, and 73.33 ± 2.47%, resp.) followed by a concurrent decrease of G_1_ phase cells (Figure 3(b)). 

### 3.4. Time-Dependent Increase of Early and Late Apoptotic Cells Induced by LIEAF

**Figure 4:**
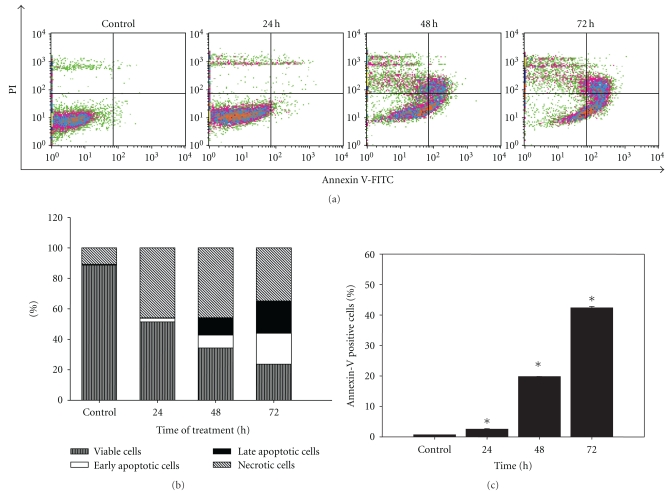
Effect of LIEAF on the externalization of phosphatidylserine in Ca Ski cells. Ca Ski cells were treated without (control) or with 500 *μ*g/mL of LIEAF for different time (24, 48, and 72 hours) and analyzed by annexin V/PI staining. (a) Flow cytometric fluorescence patterns of annexin V-PI staining. (b) Bar charts showed the percentage of distribution of viable, early apoptotic, late apoptotic and necrotic cells. (c) Results showed the percentage of annexin V positive cells. Data were means ± S.E. calculated from three individual experiments. The asterisk (*) represented significantly different from control (*P < .05).

The apoptotic potential of LIEAF was further evaluated using annexin V/PI staining. After treatment with 500 *μ*g/mL of LIEAF, Ca Ski cells demonstrated a time-dependent increase of early apoptotic cells that were stained only with annexin V, but not with PI (Figure 4(a), lower right quadrant). This early apoptotic population increased from 0.51 ± 0.02% (control) to 2.30 ± 0.13%, 8.47 ± 0.12% and 20.44 ± 0.07% after 24, 48, and 72 hours of treatment, respectively, (Figure 4(b)). Cells stained with both annexin V and PI (Figure 4(a), upper right quadrant), which represented later stage of apoptosis, were also found to increase progressively from 0.16 ± 0.00% to 0.25 ± 0.04%, 11.32 ± 0.06% and 21.91 ± 0.40% after 24, 48, and 72 hours of treatment respectively (Figure 4(b)). Taken together, we found that the percentage of annexin V positive cells (early and late apoptotic cells) increased significantly in a time-dependent manner compared to the control (Figure 4(c)). 

### 3.5. Dose- and Time-Dependent Collapse of Mitochondrial Membrane Potential Triggered by LIEAF

**Figure 5:**
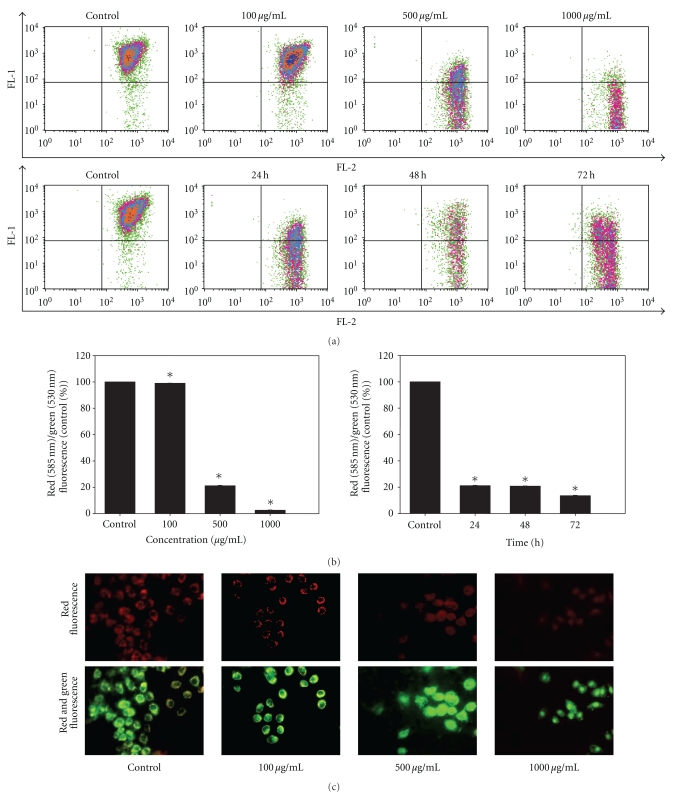
Effect of LIEAF on the change of mitochondrial membrane potential in Ca Ski cells. Ca Ski cells were incubated in the absence (control) or with varying concentrations (100, 500, and 1000 *μ*g/mL) of LIEAF for 24 hours. In another experiment, Ca Ski cells were incubated in the absence (control) or presence of 500 *μ*g/mL of LIEAF for 24, 48, and 72 hours. The cells were then stained with JC-1 and analyzed with flow cytometer. (a) Flow cytometric fluorescence patterns analysis of JC-1 staining. (b) Bar charts showed the quantitative presentation of the data as red (585 nm)/green (530 nm) fluorescence, expressed as percentage of control, indicated ratio of high/low MMP. Data presented are representative of means ± S.E. calculated from three individual experiments.  The asterisk (*) represented significantly different value from control (*P < .05). (c) Ca ski cells were treated without (control) or with indicated concentration of LIEAF for 24 hours. The cells were stained with JC-1 and analyzed by fluorescent microscope. Red fluorescence represented J-aggregate form and green fluorescence represented J-monomer form. Magnification is 400x.

Using JC-1 staining, we further analyzed the effect of LIEAF on the change of mitochondrial membrane potential. The results showed that most of the JC-1 fluorescence appeared in the upper right quadrant in the control untreated cells. However, after exposure to LIEAF, there was a shift of fluorescence signal resulting in the treated cells having lower red fluorescence than the control cells. This loss of red fluorescence increased dose- and time-dependently (Figure 5(a)). As indicated by JC-1 fluorescence ratios, LIEAF has resulted in a substantial dose- and time-dependent reduction of red/green fluorescence (Figure 5(b)). In the fluorescence microscopy analysis, the control cells showed red fluorescence image; however, the cells showed an increasingly lower red fluorescence and appeared mostly green after treated with increasing concentration of LIEAF (Figure 5(c)). 

### 3.6. Time-Dependent Induction of DNA Strand Breaks by LIEAF

**Figure 6:**
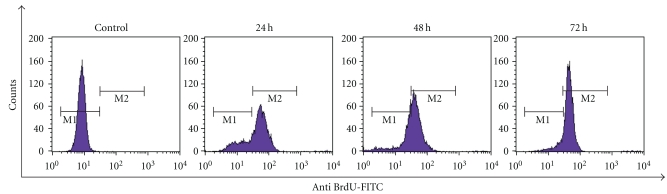
Effect of LIEAF on the cellular DNA of Ca Ski cells. Ca Ski cells were treated without (control) or with 500 *μ*g/mL of LIEAF for different time. The DNA strands breaks (DNA damage) were measured using a TUNEL assay kit as described in method. Positive TUNEL staining was shown by the M_2_ region which is the region where cells stained with FITC-conjugated anti-BrdU antibody. Histograms are representative of three separate experiments.

To further confirm the induction of apoptosis by LIEAF, DNA fragmentation was measured using TUNEL assay. As depicted in Figure 6, the Ca Ski cells showed negative TUNEL staining in the absence of LIEAF. However, cells progressively displayed positive TUNEL staining after incubation with 500 *μ*g/mL LIEAF for 24, 48, and 72 hours. 

### 3.7. Dose-Dependent Depletion of Total Glutathione (GSH) Content by LIEAF

**Figure 7:**
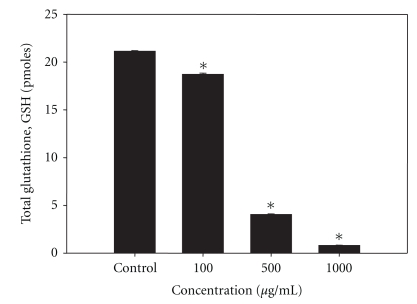
Effect of LIEAF on the intracellular GSH content in Ca Ski cells. Ca Ski cells were treated without (control) or with increasing concentration of LIEAF for 24 hours and processed for GSH determination as described in method. The GSH content was calculated as picomoles per 10^6^ cells based on a GSH standard curve. The asterisk represented significantly different value from control (*P < .05). Data were means ± S.E. calculated from three individual experiments.

In order to correlate between the intracellular GSH content and apoptosis, the intracellular total GSH content of LIEAF-treated and nontreated cells was examined. The results show that the intracellular GSH level was high in the control untreated cells and has significantly decreased in a dose-dependent manner (Figure 7) after treatment with increasing concentrations of LIEAF for 24 hours. It is noteworthy that exposure of Ca Ski cells to 500 *μ*g/mL and 1000 *μ*g/mL of LIEAF caused a 5- and 20-fold decrease of intracellular GSH content, respectively, as compared to the control (Figure 7).

### 3.8. Dose- and Time-Dependent Stimulation of DEVD-Specific Caspase-3 Activity by LIEAF

**Figure 8:**
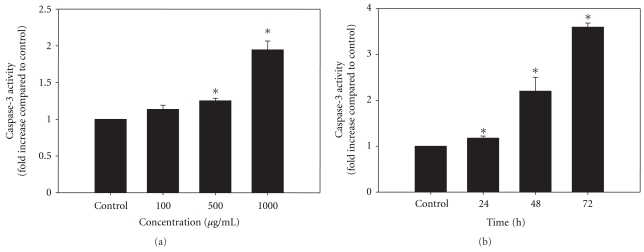
Effect of LIEAF on the activation of caspase-3 in Ca Ski cells. (a) Ca Ski cells were treated without (control) or with varying concentrations of LIEAF for 24 hours. (b) In another experiment, Ca Ski cells were treated without (control) or with 500 *μ*g/mL of LIEAF for 24, 48, and 72 hours. The DEVD-specific cleaving protease activity was then assayed in cell lysates using the caspase-3 assay kit as described in method. The activity of caspase-3 was detected by monitored the pNA liberated from Ac-DEVD-pNA, which was determined as fold increase compared to the control. The asterisk represented significantly different value from control (*P < .05). Data were means ± S.E. calculated from three individual experiments.

To determine whether LIEAF-induced apoptosis in Ca Ski cells involved the activation of caspase-3, the protease activity was examined using a colorimetric substrate specific for caspase-3, Ac-DEVD-pNA. Surprisingly, after 24 hours of treatment, LIEAF promoted a dose-dependent significant activation of DEVD-specific protease activity (Figure 8(a)). Moreover, a significant time-dependent increase in DEVD-specific protease activity was also observed when exposed to 500 *μ*g/mL LIEAF for various time periods (Figure 8(b)). The increase in activity was first observed after 24 hours and was elevated up to 2- and 3.5-fold after 48, and 72 hours, respectively. 

### 3.9. Inhibition of LIEAF-Induced Apoptosis by Caspase-3 Inhibitor

**Figure 9:**
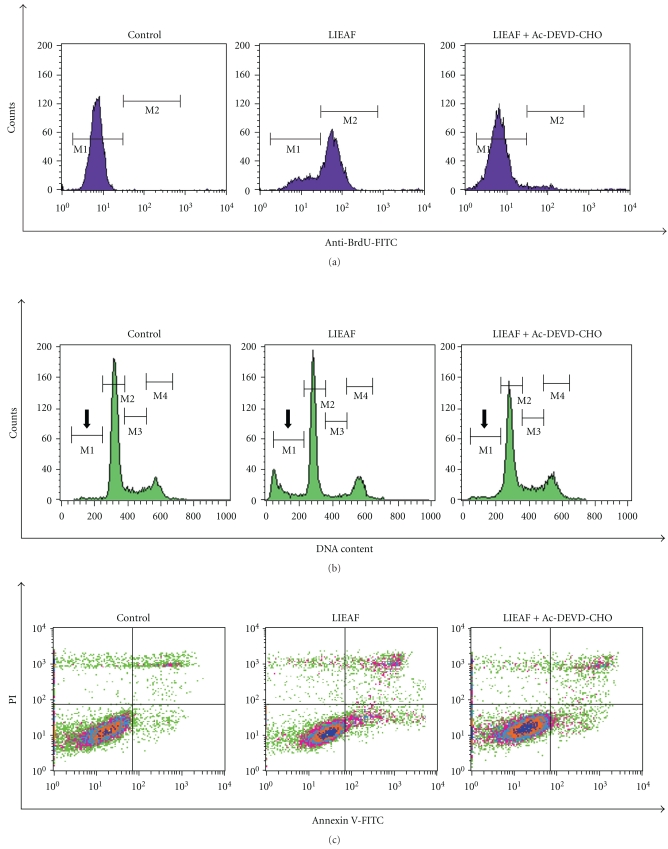

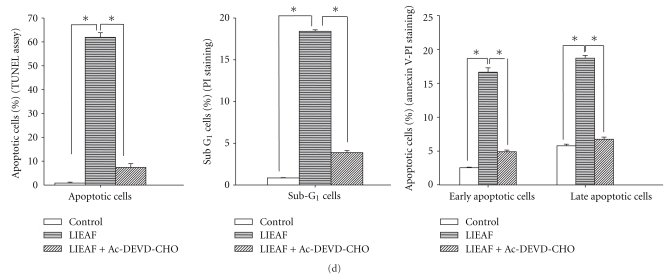
Inhibitory effect of Ac-DEVD-CHO on LIEAF-induced apoptosis in Ca Ski cells. Ca Ski cells were pretreated without or with 50 *μ*M Ac-DEVD-CHO for 1 hour prior to the treatment of 500 *μ*g/mL LIEAF for 24 hours, and then apoptosis was performed by TUNEL and PI staining. For annexin V-PI staining, cells were cultured in the absence or presence of 50 *μ*M Ac-DEVD-CHO for 1 hour before being stimulated with 500 *μ*g/mL LIEAF for 72 hours. (a) Flow cytometric analysis of TUNEL assay. (b) Histograms showing the appearance of hypodiploid sub-G_1_ cells (arrow) detected by PI staining. (c) Flow cytometric fluorescence patterns of annexin V-PI staining. (d) Summarized results for TUNEL, PI, and annexin V-PI staining. Data showed means ± S.E. of three experiments. The asterisk represented significantly different value from respective group (*P < .05).

Caspases are believed to play a pivotal role in mediating various apoptotic responses. We have previously showed that treatment with LIEAF led to the activation of caspase-3 in Ca Ski cells. To further investigate whether the caspase-3 activity is critical in the LIEAF-induced apoptosis in Ca Ski cells, we pretreated the cells with 50 *μ*M Ac-DEVD-CHO for 1 hour before being challenged with LIEAF. The inhibitory effect of Ac-DEVD-CHO on LIEAF-induced apoptosis was assessed by TUNEL, PI and annexin V-PI staining. The results from TUNEL assay have indicated that pretreatment with Ac-DEVD-CHO, followed by LIEAF treatment caused a nearly complete protection against LIEAF-induced cell death, whereby the percentage of apoptotic cells (cells which showed fragmented DNA that were labeled with anti-BrdU-FITC) was significantly decreased from 61.91 ± 1.94% to 7.38 ± 1.59% after Ac-DEVD-CHO treatment (Figures 9(a) and 9(d)). Meanwhile, the results from PI staining showed that the LIEAF-induced hypodiploid sub-G_1_ cells were almost completely abolished after the addition of Ac-DEVD-CHO, whereby it significantly decreased from 18.40 ± 0.19% to 3.88 ± 0.23% (Figures 9(b) and 9(d)). Finally, for annexin V-PI staining, Ac-DEVD-CHO was also found to attenuate the LIEAF-induced early and late apoptotic cells from 16.63 ± 0.65% and 18.70 ± 0.4% to 4.9 ± 0.25% and 6.74 ± 0.33% respectively (Figures 9(c) and 9(d)). 

## 4. Discussion

To our knowledge, this is the first study that demonstrates LIEAF-induced apoptosis in Ca Ski cells. The cytotoxic effect of the crude ethanol extract (LIEE) and fractions of *Leea indica* (LIEAF, LIHF, and LIWF) were investigated *in vitro* using MTT assay. MTT assay measured the cell viability based on the reduction of yellow tetrazolium MTT to a purple formazan dye by mitochondrial dehydrogenase enzyme. Hence, the amount of formazan produced reflected the number of metabolically active viable cells [20]. MTT results showed that *Leea indica* possessed cytotoxic effect against Ca Ski cells whereby all the extract and fractions significantly reduced formazan accumulation in a dose-dependent manner (Figure 1(a)). The Ca Ski cells varied in their sensitivity to the extract and fractions and were found most susceptible to LIEAF which demonstrated the strongest growth inhibitory effect (lowest IC_50_ value). 

Next, we aimed to investigate the sensitivity of other cell lines to LIEAF. It is notable that when the cells were treated with LIEAF at 100 *μ*g/mL and above, a significant growth suppressive effect was observed on Ca Ski, MCF 7, MDA-MB-435, KB, HEP G2 and WRL 68 cells, but, interestingly, not on Vero cells, which served as a normal cell model (Figure 1(b)). Among all the cancer cell lines, Ca Ski cells were found most susceptible to LIEAF. Therefore, Ca Ski cervical cancer cell line was selected for further studies to determine the mechanism of cell death underlying the observed growth inhibitory action. 

Since apoptotic cells exhibited some characteristic morphological features, nuclear morphological changes were assessed using DAPI staining. After 24 hours of treatment with 500 *μ*g/mL LIEAF, Ca Ski cells demonstrated signs of nuclear shrinkage and chromatin condensation (Figure 2). This indicated that Ca Ski cells underwent apoptosis when treated with LIEAF. In order to analyze whether the growth inhibition was accompanied by any alterations in cell cycle distribution, PI staining was applied to Ca Ski cells treated without and with LIEAF at different doses and time periods. The appearance of sub-G_1_ peak in the DNA histogram represented cells with hypodiploid DNA content and this sub-G_1_ population was considered as apoptotic fraction [21]. It is intriguing that LIEAF was able to induce sub-G_1_ cells dose- and time-dependently (Figure 3). These results seem to suggest that the sub-G_1_ arrest caused by LIEAF might contribute to the observed growth inhibition in Ca Ski cells. LIEAF-treated Ca Ski cells were further analyzed with annexin V/PI staining to confirm the early and late stages of apoptosis. Annexin V is a phosphatidylserine-(PS-) binding protein while PI is a DNA-binding dye and this dual staining analyzes the externalization of phosphatidylserine (PS) from the inner to the outer leaflet of membranes during the early phase of apoptosis. Therefore, Annexin V/PI can be used as a marker to identify apoptosis [22]. The time-dependent increase of early and late apoptotic cells further confirmed the induction of apoptosis by LIEAF in Ca Ski cells (Figure 4). 

Mitochondrial dysfunction such as loss of MMP is an early apoptotic event that occurs following induction of apoptosis [23]. Hence, JC-1 was performed to check the change of MMP in LIEAF-treated Ca Ski. JC-1 is a widely used dye to detect mitochondrial depolarization which occurs in the early stage of apoptosis. In the mitochondria of healthy cells, with high MMP, JC-1 forms J-aggregates and emits red fluorescence. During the onset of apoptosis, the MMP decreases and the JC-1 remains in the monomeric form and emits green fluorescence. Thus, the ratio of red to green fluorescence measures the ratio of high-to-low MMP [24]. In the control cells, JC-1 was in aggregate forms within the mitochondria, resulting in higher levels of red fluorescence, which corresponded to a polarized MMP. In contrast, in the treated cells, LIEAF dramatically reduced the formation of red fluorescent J-aggregates, indicating disruption of MMP (Figure 5(a)). The increase in dose and exposure periods of LIEAF has resulted in drastic depolarization of the MMP in Ca Ski cells. This was shown by the significant dose- and time-dependent reduction of red/green fluorescence (Figure 5(b)). These results were also observed in fluorescence microscopy analysis (Figure 5(c)) and further proved that LIEAF evoked loss of MMP in Ca Ski cells, one of the hallmarks for apoptosis [23]. 

The potential of LIEAF to induce apoptosis in Ca Ski cells was also supported by TUNEL assay which measures DNA damage in the form of fragmented DNA or DNA strand breaks by the incorporation of Br-dUTP into the exposed 3′-OH DNA ends followed by detection with fluorochrome-conjugated anti-BrdU antibody [25]. The results showed that LIEAF caused a time-dependent occurrence of DNA strand breaks in Ca Ski cells (Figure 6). We further investigated the involvement of oxidative stress in cell death by looking at the alterations of total glutathione (GSH) level. It has been reported that depletion in intracellular GSH can contribute to the onset of apoptosis, by rendering the cells more sensitive and susceptible to apoptotic agents [26]. Our results are consistent with this observation. The intracellular level of GSH was found significantly decreased following LIEAF treatment at the doses which induced apoptosis (Figure 7).

Caspases-3, a member of the cysteine proteases family, plays an important role in the execution of apoptosis. They proteolytically cleave many cellular proteins which lead to loss of cellular structure and functions, and ultimately cell death [27–29]. Therefore, we investigated the involvement of this marker of apoptosis in LIEAF-induced Ca Ski cells. In the present study, a quantitative assay was performed for caspase-3 protease activity using a commercial assay kit. After treatment with LIEAF, Ca Ski cells exhibited significant increase of caspase-3 activity in a dose- and time-dependent manner (Figure 8). Next, we investigated the possibility that the LIEAF-induced apoptosis might be due to activation of caspase-3. A caspase-3 inhibitor Ac-DEVD-CHO was used to determine whether inhibition of caspase-3 could prevent the downstream apoptotic phenotypes. Surprisingly, inhibition of caspase-3 activity by Ac-DEVD-CHO blocked LIEAF-induced apoptosis in Ca Ski cells, as shown by significant reduction of DNA fragmented cells, hypodiploid sub-G_1_ cells, early and late apoptotic cells (Figure 9). Taken together, these observations provided strong evidence that caspase-3 is crucial in LIEAF-induced apoptosis in Ca Ski cells. 

As demonstrated earlier, the apoptotic cell death responses induced by LIEAF was strongly dose- and/or time-dependent. Based on the time course analysis of apoptosis profiles, LIEAF delivered a strong apoptotic signal during early 24 hours of LIEAF treatment as characterized by a marked breakdown of MMP (Figure 5). Meanwhile, at 24 hours of treatment, cells only displayed few annexin V positive cells with less than 2-3% early and late apoptotic cells fractions (Figure 4). This observation was correlated with the cell cycle and caspase-3 analysis which showed minimal induction of sub-G_1_ cells (<20%) (Figure 3) and activation of caspase-3 (<1.2-fold) (Figure 8). However, these apoptotic responses seem to increase with time. At 72 hours, the cells demonstrated relatively higher annexin V positive cells (around 40%) (Figure 4), substantial sub-G_1_ cell accumulation (around 80%) (Figure 3) and nearly 3.5-fold increase in caspase-3 activity (Figure 8). These results were further corroborated with TUNEL assay which showed a large proportion of TUNEL positive cells at 72 hours (Figure 6). It is possible that the occurrence of DNA fragmentation (sub-G_1_ analysis) and DNA strand breaks (TUNEL assay) that were prominent at 72 hours might be attributed to the activation of intracellular caspase-3 which peaked at 72 hours. This was substantiated by the earlier study on caspase-3 inhibition whereby addition of Ac-DEVD-CHO effectively blocked those effects (Figure 9). Our data on MMP depolarization and caspase-3 induction suggested that LIEAF-induced apoptosis in Ca Ski cells might be mediated by mitochondria death pathway involving the caspase cascade. Taken together, these findings suggest a possible kinetic model in which LIEAF activated the apoptosis process in Ca Ski cells. At first, LIEAF depolarized the mitochondria, and this mitochondrial dysfunction possibly contributed to the activation of caspase-3 enzyme. At the same time, the PS residues were externalized across the plasma membrane. Then the sequential activation of caspase-3 eventually cleaved the cellular and nuclear components which caused the DNA fragmentation and DNA strands breaks. 

The earliest phytochemical work on *Leea indica* reported the isolation of *α*-tocopherol, *β*-amyrenol, *β*-amyrin and *β*-sitosteryl-*β*-D-glucopyranoside [30]. A more recent investigation reported the identification of eleven hydrocarbons, phthalic acid, palmitic acid, 1-eicosanol, solanesol, farnesol, phthalic acid esters, gallic acid, lupeol, *β*-sitosterol and ursolic acid [31]. Despite the phytochemical studies on *Leea indica*, the biological activities of this plant and its active constituents isolated from this plant have not been examined to a large extent. The evidences presented here have shown for the first time that LIEAF could inhibit the growth of cancer cells and induce apoptosis in Ca Ski cells. This warrants the need for bioactivity-guided isolation of the bioactive compound(s) in LIEAF, which is now in progress.

## 5. Conclusions

In this study, it was found that LIEAF is capable of inducing growth-suppressive and apoptosis effects in Ca Ski cells, suggesting that LIEAF possesses anti cervical cancer activity. Elaborate studies of the detailed mechanism underlying its apoptotic action are necessary. The obtained findings will definitely provide valuable information for its possible application in cervical cancer treatment in the future.
